# Supplementary material for: Affinity maturation generates pathogenic antibodies with dual reactivity to DNase1L3 and dsDNA in systemic lupus erythematosus
Source: Nat Commun. 2023 Mar 20;14:1388. doi: 10.1038/s41467-023-37083-x (PMC10027674; doi:10.1038/s41467-023-37083-x)
Supplement: Supplementary file 1 — Supplementary Information [file 41467_2023_37083_MOESM1_ESM.pdf]

Supplementary Materials for:

Affinity maturation generates pathogenic antibodies with dual reactivity to DNase1L3 and dsDNA in systemic lupus erythematosus.

Eduardo Gomez-Bañuelos, Yikai Yu, Jessica Li, Kevin S. Cashman, Merlin Paz, Maria Isabel Trejo-Zambrano, Regina Bugrovsky, Youliang Wang, Asiya Seema Chida, Cheryl Sherman-Baust, Dylan P. Ferris, Daniel W. Goldman, Erika Darrah, Michelle Petri, Iñaki Sanz, Felipe Andrade

This file contains:

Supplementary Figs. S1 to S2
Supplementary Tables S1 to S15

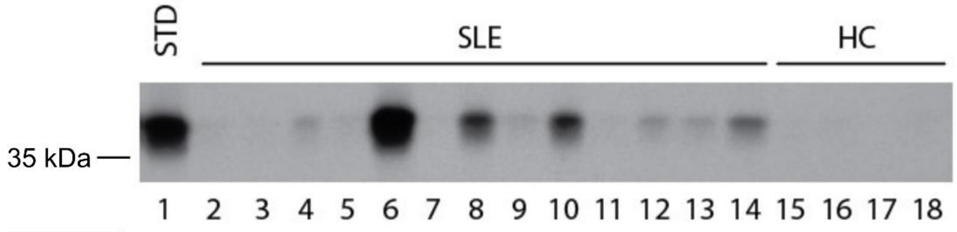

Supplementary Fig. 1. Detection of anti-DNase1L3 antibodies in sera from the SPARE cohort and healthy controls. Sera from the SPARE cohort and healthy controls (HC) were used to immunoprecipitate radiolabeled DNase1L3. Anti-DNase1L3 units were calculated by densitometry and the values were normalized to a high-titer anti-DNaseL13 serum (standard, STD). Shown is a representative radiograph including the STD, 13 SPARE, and 4 HC sera.

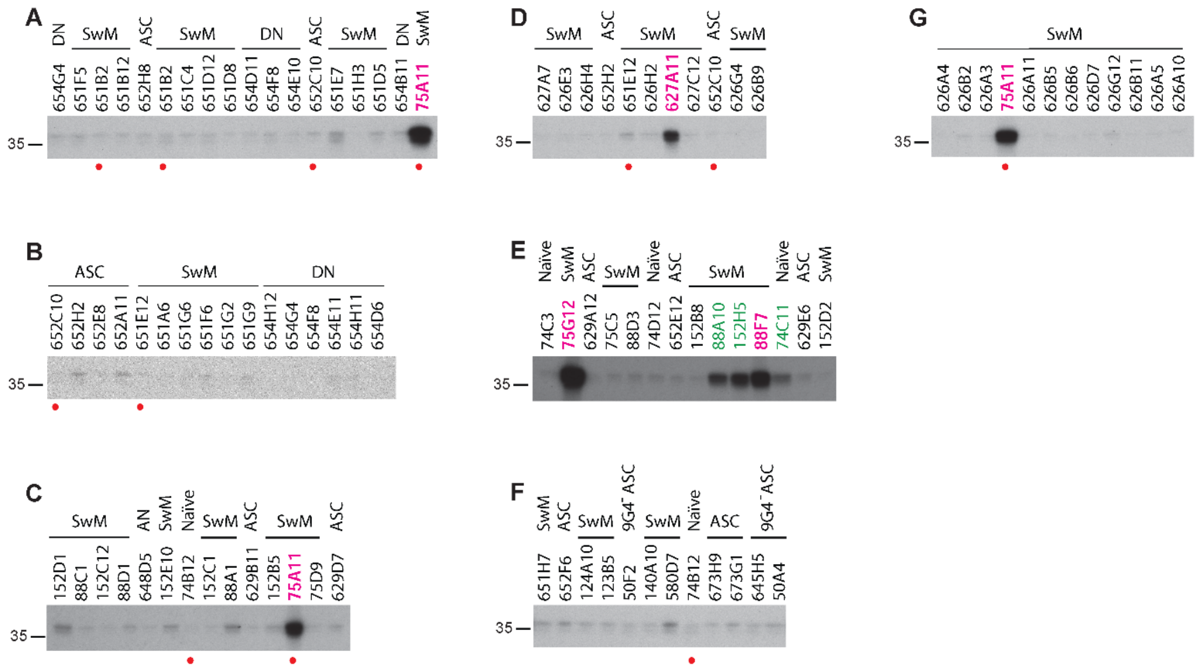

Supplementary Fig. 2. Identification of SLE patient-derived monoclonal antibodies to DNase1L3. (**A-G**). Supernatants from 293 cells expressing monoclonal antibodies generated from single B cells and antibody-secreting cells (ASCs) from SLE patients experiencing flares were screened by immunoprecipitation using radiolabeled DNase1L3. Among seven antibodies with reactivity to DNase1L3, three antibodies (panel **E**, marked in green) were not verified using affinity purified antibodies. Antibodies with confirmed reactivity to DNase1L3 are marked in pink. Antibodies marked with a red dot were unintentionally screened more than once. Except for clones 50F2, 645H5 and 50A4 (panel **F**), the rest of monoclonal antibodies are from $9G4^+$ cells. SwM, switched memory. DN, double negative.

Supplementary Table 1. Demographics and clinical characteristics of patients by anti-DNase1L3 antibody status

	**Anti-DNase1L3 Positive n = 48 (100%)**	**Anti-DNase1L3 Negative n = 110 (100%)**	**OR (95% CI)**	***P* value**
Demographic characteristics				
Female	47 (97.9%)	102 (92.7%)	3.7 (0.45,30.32)	0.278
Ethnicity				0.669
Black	18 (37.5%)	45 (40.9%)	0.9 (0.45,1.91)	
White	25 (52.1%)	58 (52.7%)	1	
Other	5 (10.4%)	7 (6.4%)	1.7 (0.48,5.73)	
Ever Smoker, n (%)	19 (39.6%)	42 (38.2%)	1.1 (0.53,2.12)	0.861
Alcohol abuse	3 (6.3%)	10 (9.1%)	0.7 (0.18,2.54)	0.756
Drugs	2 (4.2%)	7 (6.4%)	0.6 (1.13,3.20)	0.724
Clinical characteristics (Ever)				
Livedo	23 (47.9%)	33 (30%)	2.1 (1.06,4.35)	0.046
Proteinuria	29 (60.4%)	46 (41.8%)	2.1 (1.03,4.37)	0.038
Mono multiplex	2 (4.2%)	0 (0%)	N/A	0.091
Anemia	42 (87.5%)	75 (68.2%)	3.3 (1.27,8.51)	0.011
Leukopenia	27 (56.2%)	43 (39.1%)	2.0 (0.97,4.03)	0.056
Lymphopenia	26 (54.2%)	42 (38.2%)	1.9 (0.92,3.82)	0.081
Cataracts	11 (22.9%)	48 (43.6%)	0.4 (0.17,0.83)	0.020
Other arterial thrombosis	5 (10.4%)	3 (2.8%)	4.1 (0.95,20.31)	0.058
Herpes zoster	17 (35.4%)	23 (20.9%)	2.1 (0.97,4.63)	0.073
Splenomegaly	2 (4.2%)	0 (0%)	N/A	0.091
Cytotoxic use ever	38 (79.2%)	61 (55.5%)	3.0 (1.37,6.92)	0.005
Lupus anti-coagulant	23 (47.9%)	31 (28.2%)	2.3 (1.15,4.81)	0.019
Anti-cardiolipin	39 (81.2%)	65 (59.1%)	3.0 (1.3,7.05)	0.010
Anti-B2 Glycoprotein	22 (45.8%)	27 (25%)	2.5 (1.21,5.35)	0.015
Anti-Sm	11 (22.9%)	21 (19.1%)	1.3 (0.51,2.92)	0.668
FP-RPR	7 (14.6%)	11 (10%)	1.5 (0.49,4.37)	0.423
ANA	48 (100%)	109 (99.1%)	N/A	1.000
Anti-dsDNA	41 (85.4%)	58 (52.7%)	5.2 (2.09,13.54)	0.0001
Anti-Ro	22 (45.8%)	27 (24.5%)	2.6 (1.24,5.48)	0.009
Anti-La	9 (18.8%)	15 (13.6%)	1.5 (0.54,3.69)	0.471
Anti-RNP	18 (37.5%)	24 (21.8%)	2.1 (0.99,4.71)	0.051
Low CH50	15 (31.2%)	8 (7.3%)	5.7 (2.17,14.85)	0.0003
Low C3	39 (81.2%)	48 (43.6%)	5.5 (2.44,13.11)	< 0.0001
Low C4	33 (68.8%)	39 (35.5%)	4.0 (1.85,8.46)	0.0001
Low complement C3/C4	39 (81.2%)	56 (50.9%)	4.1 (1.82,9.77)	0.0004
Coombs	12 (25%)	13 (11.8%)	2.5 (0.96,6.24)	0.056
Elevated ESR	37 (77.1%)	82 (74.5%)	1.2 (0.49,2.73)	0.842
At time of visit				
SELENA-SLEDAI, median (IQR)	2.5 (2,4)	1 (0,2)	NA	<0.0001
PGA, median (IQR)	0.5 (0,1)	0.5 (0, 0.5)	NA	0.537
C3, mean (SD)	110.02 (36.95)	135.63 (35.75)	NA	0.0001
C4, median (IQR)	18 (12, 27)	24.5 (20, 32)	NA	0.0003
Anti-dsDNA, median (IQR)	10 (0, 80)	0 (0,0)	NA	<0.0001
**SLEDAI (At time of visit)**				
CNS	0(0%)	1 (0.9%)	NA	1.000
Vascular	1 (2.2%)	2 (1.9%)	1.2 (0.10,12.98)	1.000
Renal	3 (6.5%)	5 (4.7%)	1.4 (0.32,6.11)	0.698
Musculoskeletal	4 (8.7%)	5 (4.7%)	1.9 (0.49,7.45)	0.454
Immunological	27 (58.7%)	16 (15.0%)	7.6 (3.47,16.45)	<0.0001
Skin	17 (37.0%)	32 (29.9%)	1.3 (0.65,2.75)	0.451
Serositis	1 (2.2%)	2 (1.9%)	1.2 (0.10,13.27)	1.000
Hematology	2 (4.4%)	1 (0.9%)	4.7 (0.41,53.6)	0.215
Constitutional	0 (0%)	0 (0%)	NA	NA

OR: Odds ratio. CI: confidence interval. ESR: Erythrocyte sedimentation rate. SELENA-SLEDAI: Safety of Estrogens in Lupus National Assessment study-SLE disease activity index. PGA:

physician global assessment. CNS: central nervous system. Comparisons of categorical variables were done using a two-sided Fisher's exact test. Comparisons of continuous variables were done using a two-sided Student's T test.

Supplementary Table 2. Multivariate predictors of cytotoxic drugs and prednisone use ‘at time of visit’ in patients with SLE.

	**Cytotoxic drug use**		**Prednisone use**	
Variable	OR (95% CI)	*P* value	OR (95% CI)	*P* value
Anti-DNase1L3	4.6 (2.16 – 9.89)	<0.001	2.6 (1.23 – 5.42)	0.013
SLEDAI	1.1 (0.95 – 1.22)	0.263	1.1 (0.99 – 1.28)	0.053

Odds ratios and 95% Confidence Iintervals (CI) were estimated using multivariate logistic regression, adjusted for anti-DNase1L3 status and SLEDAI. *P* values are two-sided.

Supplementary Table 3. Differentially active modules in anti-DNase1L3 positive SLE patients *vs*. healthy controls.

Module	Description	logFC	Average Expr	t	Adjusted *P*. Value	B
DC.M1.2	Interferon	7.95	1.77	6.40	5.73E-08	11.51
DC.M3.4	Interferon	5.64	3.81	7.15	1.84E-09	15.49
DC.M5.12	Interferon	3.36	6.17	6.78	9.82E-09	13.48
DC.M7.16	Myeloid lineage	2.42	2.84	5.96	4.40E-07	9.34
DC.M5.15	Myeloid lineage	1.76	-2.59	2.56	0.035719236	-3.42
DC.M7.21	Other/Undetermined	1.48	1.84	5.37	4.98E-06	6.57
DC.M7.15	Myeloid lineage	1.46	5.06	3.27	0.008448325	-1.49
DC.M6.3	RNA metabolism/processing	1.41	5.84	3.72	0.002339732	-0.03
DC.M7.10	Myeloid lineage	1.24	1.81	2.52	0.039416595	-3.53
DC.M8.48	RNA metabolism/processing	1.22	1.09	5.53	3.17E-06	7.27
DC.M8.60	Myeloid lineage	1.14	1.76	4.62	8.87E-05	3.32
DC.M7.2	Other/Undetermined	1.13	3.91	3.23	0.009064687	-1.60
DC.M8.18	Inflammation	1.07	-2.34	3.18	0.010174195	-1.76
DC.M7.34	Lymphoid lineage	1.07	3.84	5.16	1.09E-05	5.61
DC.M7.29	Myeloid lineage	0.98	1.24	3.38	0.006052795	-1.14
DC.M9.22	Other/Undetermined	0.97	1.70	3.01	0.014643216	-2.24
DC.M5.2	Inflammation	0.93	4.47	3.12	0.011597499	-1.94
DC.M9.24	Interferon	0.87	1.18	3.45	0.004913589	-0.91
DC.M8.2	Other/Undetermined	0.85	4.80	4.78	5.02E-05	3.98
DC.M8.69	Other/Undetermined	0.83	1.39	4.61	8.87E-05	3.30
DC.M9.29	Apoptosis	0.74	0.71	2.51	0.039481302	-3.54
DC.M9.1	RNA metabolism/processing	0.66	-2.64	4.81	4.77E-05	4.11
DC.M9.7	Other/Undetermined	0.42	-1.79	2.61	0.032740512	-3.31
DC.M8.43	Other/Undetermined	-0.33	2.98	-2.88	0.018640992	-2.60
DC.M9.37	Other/Undetermined	-0.36	1.85	-2.67	0.029076506	-3.15
DC.M9.13	Other/Undetermined	-0.38	-1.31	-2.63	0.031774271	-3.25
DC.M9.18	Other/Undetermined	-0.43	1.55	-2.91	0.017952989	-2.52
DC.M9.12	Other/Undetermined	-0.47	0.68	-2.57	0.035271025	-3.39
DC.M7.8	RNA metabolism/processing	-0.48	0.61	-2.42	0.04988711	-3.76
DC.M4.3	RNA metabolism/processing	-0.48	8.34	-2.75	0.024250822	-2.94
DC.M8.6	Other/Undetermined	-0.50	-0.74	-2.78	0.023639798	-2.86
DC.M8.22	Other/Undetermined	-0.51	-1.21	-2.95	0.016718509	-2.41
DC.M2.1	Other/Undetermined	-0.54	1.65	-3.13	0.011462212	-1.90
DC.M9.16	Other/Undetermined	-0.61	-0.35	-2.88	0.018640992	-2.61
DC.M9.23	Other/Undetermined	-0.65	-1.65	-3.91	0.00126441	0.62
DC.M9.28	Other/Undetermined	-0.66	-3.15	-2.94	0.016999169	-2.45
DC.M8.32	Lymphoid lineage	-0.68	0.01	-2.87	0.018819028	-2.64
DC.M4.8	RNA metabolism/processing	-0.69	1.38	-2.99	0.015141601	-2.30
DC.M8.5	RNA metabolism/processing	-0.71	-2.69	-2.70	0.027437819	-3.08
DC.M8.44	Other/Undetermined	-0.73	1.74	-2.75	0.024250822	-2.95

DC.M8.16	Other/Undetermined	-0.78	-2.28	-5.27	7.28E-06	6.09
DC.M7.19	RNA metabolism/processing	-0.84	0.43	-2.77	0.023655249	-2.88
DC.M7.26	Mitochondria	-1.04	2.50	-5.49	3.21E-06	7.11
DC.M7.17	Lymphoid lineage	-1.10	-1.03	-3.59	0.003210095	-0.48
DC.M7.6	RNA metabolism/processing	-1.18	-5.64	-3.64	0.002884011	-0.29
DC.M6.2	Other/Undetermined	-1.21	0.53	-4.27	0.000321753	1.96
DC.M7.12	RNA metabolism/processing	-1.30	-4.43	-3.71	0.002339732	-0.05
DC.M5.6	Mitochondria	-1.36	-2.68	-2.63	0.031774271	-3.27
DC.M3.5	Cell cycle	-1.50	-9.33	-3.22	0.009064687	-1.62
DC.M7.13	RNA metabolism/processing	-1.64	-2.58	-4.36	0.0002436	2.28
DC.M6.4	RNA metabolism/processing	-1.79	0.63	-7.60	2.78E-10	17.98
DC.M5.10	Mitochondria	-1.84	-5.59	-3.08	0.012265305	-2.05
DC.M3.6	Lymphoid lineage	-2.17	-1.64	-3.09	0.01213926	-2.01
DC.M5.5	Lymphoid lineage	-2.29	0.27	-3.61	0.003126304	-0.41

Supplementary Table 4. Differentially active modules in anti-DNase1L3 negative SLE patients *vs*. healthy controls.

Module	Description	logFC	Average Expression	t	Adjusted *P*. value	B
DC.M1.2	Interferon	4.01	1.77	3.52	0.009875473	-0.57
DC.M3.4	Interferon	2.48	3.81	3.42	0.012553444	-0.89
DC.M5.12	Interferon	1.54	6.17	3.38	0.013405332	-1.04
DC.M6.3	RNA metabolism/processing	1.05	5.84	3.04	0.02751127	-2.04
DC.M8.48	RNA metabolism/processing	0.83	1.09	4.09	0.00210667	1.39
DC.M9.25	Other/Undetermined	0.81	0.61	3.83	0.003735567	0.46
DC.M8.60	Myeloid lineage	0.73	1.76	3.21	0.019482586	-1.53
DC.M7.34	Lymphoid lineage	0.73	3.84	3.82	0.003735567	0.43
DC.M9.1	RNA metabolism/processing	0.65	-2.64	5.16	5.37E-05	5.69
DC.M8.69	Other/Undetermined	0.54	1.39	3.30	0.015883252	-1.27
DC.M8.2	Other/Undetermined	0.52	4.80	3.19	0.019664361	-1.60
DC.M8.37	Other/Undetermined	-0.57	3.91	-3.03	0.02751127	-2.07
DC.M9.16	Other/Undetermined	-0.58	-0.35	-3.02	0.02751127	-2.08
DC.M2.1	Other/Undetermined	-0.62	1.65	-3.95	0.003021229	0.89
DC.M9.23	Other/Undetermined	-0.67	-1.65	-4.43	0.000678785	2.66
DC.M7.26	Mitochondria	-0.77	2.50	-4.43	0.000678785	2.66
DC.M6.4	RNA metabolism/processing	-1.40	0.63	-6.47	1.52E-07	11.92

Supplementary Table 5. Differentially active modules on anti-DNase1L3 positive *vs*. negative patients with SLE.

Module	Description	logFC	Average expression	t	Adjusted *P*. value	B
DC.M1.2	Interferon	3.93	1.77	5.03	3.97E-05	5.12
DC.M3.4	Interferon	3.16	3.81	6.37	2.61E-07	11.40
DC.M5.12	Interferon	1.82	6.17	5.85	1.97E-06	8.82
DC.M7.16	Myeloid lineage	1.46	2.84	5.73	2.38E-06	8.26
DC.M5.15	Myeloid lineage	1.44	-2.59	3.33	0.007846893	-1.17
DC.M4.2	Inflammation	1.12	2.78	2.53	0.041085413	-3.36
DC.M6.6	Apoptosis	1.01	0.29	3.87	0.002011952	0.59
DC.M6.13	Apoptosis	0.93	2.10	3.42	0.006968963	-0.88
DC.M7.21	Other/Undetermined	0.90	1.84	5.22	2.09E-05	5.93
DC.M7.10	Myeloid lineage	0.85	1.81	2.73	0.029237076	-2.86
DC.M5.1	Inflammation	0.81	12.22	2.43	0.04885988	-3.58
DC.M4.6	Inflammation	0.77	10.61	2.72	0.029237076	-2.88
DC.M7.15	Myeloid lineage	0.74	5.06	2.63	0.034895318	-3.11
DC.M5.2	Inflammation	0.59	4.47	3.13	0.011960732	-1.77
DC.M9.24	Interferon	0.56	1.18	3.52	0.00588123	-0.56
DC.M7.29	Myeloid lineage	0.54	1.24	2.93	0.019305229	-2.31
DC.M8.60	Myeloid lineage	0.41	1.76	2.65	0.034597115	-3.05
DC.M8.48	RNA metabolism/processing	0.39	1.09	2.81	0.024258362	-2.64
DC.M7.34	Lymphoid lineage	0.34	3.84	2.62	0.034895318	-3.12
DC.M8.2	Other/Undetermined	0.33	4.80	2.94	0.019305229	-2.28
DC.M8.69	Other/Undetermined	0.28	1.39	2.52	0.041209835	-3.38
DC.M9.14	Other/Undetermined	0.28	-0.97	2.81	0.024258362	-2.65
DC.M9.13	Other/Undetermined	-0.24	-1.31	-2.60	0.035347589	-3.18
DC.M8.43	Other/Undetermined	-0.26	2.98	-3.59	0.005061839	-0.36
DC.M2.2	Cell cycle	-0.29	-1.41	-3.27	0.009142776	-1.35
DC.M9.12	Other/Undetermined	-0.33	0.68	-2.90	0.019907533	-2.40
DC.M8.54	RNA metabolism/processing	-0.36	0.24	-2.91	0.019907533	-2.39
DC.M8.6	Other/Undetermined	-0.37	-0.74	-3.24	0.009266393	-1.43
DC.M4.8	RNA metabolism/processing	-0.38	1.38	-2.60	0.035347589	-3.19
DC.M6.4	RNA metabolism/processing	-0.39	0.63	-2.64	0.034895318	-3.08
DC.M8.16	Other/Undetermined	-0.40	-2.28	-4.31	0.000621304	2.22
DC.M8.14	Other/Undetermined	-0.46	-0.97	-3.46	0.006450071	-0.76
DC.M8.15	Other/Undetermined	-0.47	-3.71	-4.05	0.001384467	1.25
DC.M6.2	Other/Undetermined	-0.49	0.53	-2.73	0.029237076	-2.86
DC.M9.5	Other/Undetermined	-0.49	-1.24	-2.93	0.019305229	-2.32
DC.M7.6	RNA metabolism/processing	-0.50	-5.64	-2.47	0.045082647	-3.49
DC.M8.8	Apoptosis	-0.55	-0.47	-3.35	0.007665713	-1.10
DC.M7.20	Lymphoid lineage	-0.56	-0.98	-2.61	0.035347589	-3.16
DC.M8.32	Lymphoid lineage	-0.57	0.01	-3.86	0.002011952	0.56

DC.M9.2	Myeloid lineage	-0.58	-5.14	-4.21	0.000824286	1.84
DC.M7.18	Lymphoid lineage	-0.58	-2.07	-2.85	0.022738635	-2.54
DC.M8.25	Lymphoid lineage	-0.67	1.55	-3.20	0.010172074	-1.55
DC.M7.25	Lymphoid lineage	-0.67	-4.58	-3.01	0.016882333	-2.11
DC.M6.7	Other/Undetermined	-0.74	-5.32	-2.50	0.04280179	-3.43
DC.M5.8	RNA metabolism/processing	-0.75	-4.24	-3.26	0.009142776	-1.39
DC.M7.17	Lymphoid lineage	-0.75	-1.03	-3.91	0.001956566	0.74
DC.M5.11	Lymphoid lineage	-0.78	0.20	-3.38	0.007656499	-1.01
DC.M7.5	RNA metabolism/processing	-0.81	-4.23	-3.35	0.007665713	-1.10
DC.M7.12	RNA metabolism/processing	-0.88	-4.43	-3.98	0.00162993	1.00
DC.M7.13	RNA metabolism/processing	-1.11	-2.58	-4.68	0.000154482	3.67
DC.M4.10	Lymphoid lineage	-1.20	-3.04	-2.55	0.038815225	-3.29
DC.M4.7	Cell cycle	-1.32	-3.74	-3.17	0.010887101	-1.65
DC.M5.5	Lymphoid lineage	-1.40	0.27	-3.49	0.006285316	-0.68

Supplementary Table 6. Multivariable predictors of module M1.2 activity on SLE patients.

Dependent variable: Module M1.2

	Model 1		Model 2	
**Variable**	**Estimate**	***P* value**	**Estimate**	***P* value**
Anti-DNase1L3	2.951	0.002	2.746	0.002
Anti-DNA	2.629	0.008	2.584	0.007
Neutrophil count	-0.002	0.313		
cSLEDAI	0.240	0.138		
Hydroxychloroquine	-2.64	0.006	-2.521	0.008
Immunosuppressors	-0.007	0.993		
Prednisone use	-0.647	0.444		

Dependent variables were input as individual normalized enrichment scores (NES) calculated by ssGSEA. Neutrophil count represents the MCP score on arbitrary units. cSLEDAI, clinical SLEDAI score was calculated by subtraction of the increased-DNA binding and low complement score from the SELENA-SLEDAI score. Hydroxychloroquine, immunosuppressors, and prednisone use were entered as dummy variables coded as 0 when absent or 1 when present. Two-sided *P* values are shown.

Supplementary Table 7. Multivariable predictors of module M3.4 activity on SLE patients.

Dependent variable: Module M3.4

	Model 1		Model 2	
Variable	Estimate	*P* value	Estimate	*P* value
Anti-DNase1L3	2.424	<0.001	2.295	<0.001
Anti-DNA	1.872	0.003	1.911	0.002
Neutrophil count	-0.0003	0.806		
cSLEDAI	0.203	0.046		
Hydroxychloroquine	-1.781	0.003	-1.734	0.003
Immunosuppressors	-0.190	0.528		
Prednisone use	-0.414	0.0.433		

Dependent variables were input as individual normalized enrichment scores (NES) calculated by ssGSEA. Neutrophil count represents the MCP score on arbitrary units. cSLEDAI, clinical SLEDAI score was calculated by subtraction of the increased-DNA binding and low complement score from the SELENA-SLEDAI score. Hydroxychloroquine, immunosuppressors, and prednisone use were entered as dummy variables coded as 0 when absent or 1 when present. Two-sided *P* values are shown.

Supplementary Table 8. Multivariable predictors of module M5.12 activity on SLE patients.

Dependent variable: Module M5.12

	Model 1		Model 2	
Variable	Estimate	*P* value	Estimate	*P* value
Anti-DNase1L3	1.145	0.001	1.104	0.001
Anti-DNA	1.136	0.002	1.156	0.002
Neutrophil count	0.003	0.0001	0.002	0.001
cSLEDAI	0.082	0.178		
Hydroxychloroquine	-1.107	0.002	-1.107	0.002
Immunosuppressors	-0.179	0.572		
Prednisone use	-0.051	0.871		

Dependent variables were input as individual normalized enrichment scores (NES) calculated by ssGSEA. Neutrophil count represents the MCP score on arbitrary units. cSLEDAI, clinical SLEDAI score was calculated by subtraction of the increased-DNA binding and low complement score from the SELENA-SLEDAI score. Hydroxychloroquine, immunosuppressors, and prednisone use were entered as dummy variables coded as 0 when absent or 1 when present. Two-sided *P* values are shown.

Supplementary Table 9. Multivariable predictors of module M7.16 activity on SLE patients.

Dependent variable: Module M7.16

	Model 1		Model 2	
Variable	Estimate	*P* value	Estimate	*P* value
Anti-DNase1L3	0.717	0.002	0.754	<0.001
Anti-DNA	0.764	0.002	0.823	<0.001
Neutrophil count	0.005	<0.001	0.004	<0.001
cSLEDAI	0.026	0.509		
Hydroxychloroquine	-0.176	0.444		
Immunosuppressors	0.246	0.231		
Prednisone use	-0.252	0.221		

Dependent variables were input as individual normalized enrichment scores (NES) calculated by ssGSEA. Neutrophil count represents the MCP score on arbitrary units. cSLEDAI, clinical SLEDAI score was calculated by subtraction of the increased-DNA binding and low complement score from the SELENA-SLEDAI score. Hydroxychloroquine, immunosuppressors, and prednisone use were entered as dummy variables coded as 0 when absent or 1 when present. Two-sided *P* values are shown.

Supplementary Table 10. Multivariable predictors of module M5.15 activity on SLE patients.

Dependent variable: Module M5.15

	Model 1		Model 2	
Variable	Estimate	*P* value	Estimate	*P* value
Anti-DNase1L3	0.401	0.377		
Anti-DNA	0.487	0.314	1.212	0.0103
Neutrophil count	0.005	<0.0001		
cSLEDAI	0.205	0.0104		
Hydroxychloroquine	-0.764	0.10		
Immunosuppressors	0.096	0.816		
Prednisone use	0.9183	0.0284	1.823	<0.001

Dependent variables were input as individual normalized enrichment scores (NES) calculated by ssGSEA. Neutrophil count represents the MCP score on arbitrary units. cSLEDAI, clinical SLEDAI score was calculated by subtraction of the increased-DNA binding and low complement score from the SELENA-SLEDAI score. Hydroxychloroquine, immunosuppressors, and prednisone use were entered as dummy variables coded as 0 when absent or 1 when present. Two-sided *P* values are shown.

Supplementary Table 11. SLE patient-derived monoclonal antibodies

Clone	Sorted Population
**140A10**	9G4+ SWM (CD27+ IGD-)
**152B5**	9G4+ SWM (CD27+ IGD-)
**152B8**	9G4+ SWM (CD27+ IGD-)
**152C1**	9G4+ SWM (CD27+ IGD-)
**152C12**	9G4+ SWM (CD27+ IGD-)
**152D1**	9G4+ SWM (CD27+ IGD-)
**152D2**	9G4+ SWM (CD27+ IGD-)
**152E10**	9G4+ SWM (CD27+ IGD-)
**152H5**	9G4+ SWM (CD27+ IGD-)
**580D7**	9G4+ SWM (CD27+ IGD-)
**626A10**	9G4+ SWM (CD27+ IGD-)
**626A11**	9G4+ SWM (CD27+ IGD-)
**626A3**	9G4+ SWM (CD27+ IGD-)
**626A4**	9G4+ SWM (CD27+ IGD-)
**626A5**	9G4+ SWM (CD27+ IGD-)
**626B11**	9G4+ SWM (CD27+ IGD-)
**626B2**	9G4+ SWM (CD27+ IGD-)
**626B5**	9G4+ SWM (CD27+ IGD-)
**626B6**	9G4+ SWM (CD27+ IGD-)
**626B9**	9G4+ SWM (CD27+ IGD-)
**626D7**	9G4+ SWM (CD27+ IGD-)
**626E3**	9G4+ SWM (CD27+ IGD-)
**626G12**	9G4+ SWM (CD27+ IGD-)
**626G4**	9G4+ SWM (CD27+ IGD-)
**626H2**	9G4+ SWM (CD27+ IGD-)
**626H4**	9G4+ SWM (CD27+ IGD-)
**627A11**	9G4+ SWM (CD27+ IGD-)
**627A7**	9G4+ SWM (CD27+ IGD-)
**627C12**	9G4+ SWM (CD27+ IGD-)
**651A6**	9G4+ SWM (CD27+ IGD-)
**651B12**	9G4+ SWM (CD27+ IGD-)
**651B2**	9G4+ SWM (CD27+ IGD-)
**651C4**	9G4+ SWM (CD27+ IGD-)
**651D12**	9G4+ SWM (CD27+ IGD-)
**651D5**	9G4+ SWM (CD27+ IGD-)
**651D8**	9G4+ SWM (CD27+ IGD-)
**651E12**	9G4+ SWM (CD27+ IGD-)
**651E7**	9G4+ SWM (CD27+ IGD-)
**651F5**	9G4+ SWM (CD27+ IGD-)
**651F6**	9G4+ SWM (CD27+ IGD-)
**651G2**	9G4+ SWM (CD27+ IGD-)
**651G6**	9G4+ SWM (CD27+ IGD-)
**651G9**	9G4+ SWM (CD27+ IGD-)
**651H3**	9G4+ SWM (CD27+ IGD-)
**651H7**	9G4+ SWM (CD27+ IGD-)
**75A11**	9G4+ SWM (CD27+ IGD-)
**75C5**	9G4+ SWM (CD27+ IGD-)
**75D9**	9G4+ SWM (CD27+ IGD-)
**75G12**	9G4+ SWM (CD27+ IGD-)
**88A1**	9G4+ SWM (CD27+ IGD-)
**88A10**	9G4+ SWM (CD27+ IGD-)
**88C1**	9G4+ SWM (CD27+ IGD-)
**88D1**	9G4+ SWM (CD27+ IGD-)
**88D3**	9G4+ SWM (CD27+ IGD-)
**88F7**	9G4+ SWM (CD27+ IGD-)
**123B5**	9G4+ ASC(CD27hi CD38hi)
**124A10**	9G4+ ASC(CD27hi CD38hi)
**629A12**	9G4+ ASC(CD27hi CD38hi)
**629B11**	9G4+ ASC(CD27hi CD38hi)
**629D7**	9G4+ ASC(CD27hi CD38hi)
**629E6**	9G4+ ASC(CD27hi CD38hi)
**652A11**	9G4+ ASC(CD27hi CD38hi)
**652C10**	9G4+ ASC(CD27hi CD38hi)
**652E12**	9G4+ ASC(CD27hi CD38hi)
**652E8**	9G4+ ASC(CD27hi CD38hi)
**652F6**	9G4+ ASC(CD27hi CD38hi)
**652H2**	9G4+ ASC(CD27hi CD38hi)
**652H8**	9G4+ ASC(CD27hi CD38hi)
**673G1**	9G4+ ASC(CD27hi CD38hi)
**673H9**	9G4+ ASC(CD27hi CD38hi)
**648D5**	9G4+ acN (CD27- IgD+ CD11c+)
**654B11**	9G4+ DN (CD27- IGD-)
**654D11**	9G4+ DN (CD27- IGD-)
**654D6**	9G4+ DN (CD27- IGD-)
**654E10**	9G4+ DN (CD27- IGD-)
**654E11**	9G4+ DN (CD27- IGD-)
**654F8**	9G4+ DN (CD27- IGD-)
**654G4**	9G4+ DN (CD27- IGD-)
**654H11**	9G4+ DN (CD27- IGD-)
**654H12**	9G4+ DN (CD27- IGD-)
**74B12**	9G4+ Naïve (CD27- IgD+)
**74C3**	9G4+ Naïve (CD27- IgD+)
**74C11**	9G4+ Naïve (CD27- IgD+)
**74D12**	9G4+ Naïve (CD27- IgD+)
**50A4**	9G4- ASC(CD27hi CD38hi)
**50F2**	9G4- ASC(CD27hi CD38hi)
**646H5**	9G4- ASC(CD27hi CD38hi)
SWM, switched memory; ASC, antibody-secreting cells; DN, double negative.	

Supplementary Table 12. Ig gene usage, mutation number and CDR3 amino acid sequences of monoclonal antibody C4.

Clone	Chain	V	D	J	Mutations	Missense mutations	CDR3 sequence
C4	Heavy	HV3-49*03	HD2-2*01	HJ4*02	11	4	TRNRPGYCSGTSCCLD
	Light	KV2-28*01		KJ4*01	7	4	MQALQIPQT

Supplementary Table 13. EC_{50} of anti-DNase1L3 monoclonal antibodies and their germline variants against DNase1L3, dsDNA, and cardiolipin.

**Antigen**	**75G12**	**75A11**	**627A11**	**88F7**	**75G12/A11$_{GL}$**	**627A11$_{GL}$**	**88F7$_{GL}$**
DNase1L3	13.5 ± 2.90	38.5 ± 2.44	53.9 ± 12.37	247.1 + 5.52	119.6 + 75.58	55.1 ± 33.38	23.4 ± 5.52
dsDNA	387.2 ± 112.35	332.8 ± 159.15	43.0 ± 9.25	63.0 ± 4.65	1031.0 ± 8.49	794.0 ± 130.32	84.9 ± 4.65
Cardiolipin	164.2 ± 51.19	NA	270.1 ± 24.89	45.7 ± 7.71	NA	NA	339.8 ± 7.71

Values are expressed as mean ± standard deviation of the EC_{50} in [nM]. Experiments were performed in duplicate.

Supplementary Table 14. EC_{50} of anti-dsDNA monoclonal antibodies and their germline variants against DNase1L3, dsDNA, and cardiolipin.

**Antigen**	**32.B9**	**33.C9**	**33.H11**	**RH14**	**32.B9$_{GL}$**	**33.C9$_{GL}$**	**33.H11/RH14$_{GL}$**
DNase1L3	NA	0.7 ± 0.28	0.3 ± 0.3	0.4 ± 0.21	NA	NA	NA
dsDNA	20.3 ± 6.06	16 ± 9.56	16.3 ± 6.48	22.1 ± 0.17	57 ± 12.49	NA	NA
Cardiolipin	NA	NA	NA	NA	NA	NA	NA

Values are expressed as mean ± standard deviation of the EC_{50} in [nM]. Experiments were performed in duplicate.

Supplementary Table 15. Oligonucleotide sequences

**Name**	**Sequence (5' to 3')**
DNase1L3-F	GAATTCGCCATGAGGATCTGCTCCTTCAA
DNase1L3-R	GCGGCCGCGGTCTAGGAGCGTTTGCTCTTTGTTTT
DHApo1	AGCACTCTCGAGCCTCTCACCGCA
DHApo2	TGCGGTGAGAGG
