# Supplementary material for: Affinity maturation generates pathogenic antibodies with dual reactivity to DNase1L3 and dsDNA in systemic lupus erythematosus
Source: Nat Commun. 2023 Mar 20;14:1388. doi: 10.1038/s41467-023-37083-x (PMC10027674; doi:10.1038/s41467-023-37083-x)
Supplement: Supplementary file 3 — Reporting Summary [file 41467_2023_37083_MOESM3_ESM.pdf]

nature portfolio

Corresponding author(s): Felipe Andrade
Last updated by author(s): 2023/02/20

Reporting Summary

Nature Portfolio wishes to improve the reproducibility of the work that we publish. This form provides structure for consistency and transparency in reporting. For further information on Nature Portfolio policies, see our Editorial Policies and the Editorial Policy Checklist.

Statistics

For all statistical analyses, confirm that the following items are present in the figure legend, table legend, main text, or Methods section.

n/a	Confirmed	
☐	☒	The exact sample size (n) for each experimental group/condition, given as a discrete number and unit of measurement
☒	☐	A statement on whether measurements were taken from distinct samples or whether the same sample was measured repeatedly
☐	☒	The statistical test(s) used AND whether they are one- or two-sided<br>*Only common tests should be described solely by name; describe more complex techniques in the Methods section.*
☐	☒	A description of all covariates tested
☐	☒	A description of any assumptions or corrections, such as tests of normality and adjustment for multiple comparisons
☐	☒	A full description of the statistical parameters including central tendency (e.g. means) or other basic estimates (e.g. regression coefficient) AND variation (e.g. standard deviation) or associated estimates of uncertainty (e.g. confidence intervals)
☐	☒	For null hypothesis testing, the test statistic (e.g. F, t, r) with confidence intervals, effect sizes, degrees of freedom and P value noted<br>*Give P values as exact values whenever suitable.*
☒	☐	For Bayesian analysis, information on the choice of priors and Markov chain Monte Carlo settings
☒	☐	For hierarchical and complex designs, identification of the appropriate level for tests and full reporting of outcomes
☐	☒	Estimates of effect sizes (e.g. Cohen's d, Pearson's r), indicating how they were calculated

Our web collection on statistics for biologists contains articles on many of the points above.

Software and code

Policy information about availability of computer code

Data collection	No software was used for data collection. The study included the analysis of stored samples and clinical data from the "Study of biological Pathways, Disease Activity and Response markers in patients with Systemic Lupus Erythematosus" (SPARE) published in 2015 (Ref. 61).
Data analysis	The following software and packages were used to conduct the analyses: R (4.2.1), Bioconductor (3.15.2), Rstudio (2022.07.1), Exact2x2 (1.6.6), ssGSEA2.0 (2.0), limma(3.52.3), Complex heatmap (2.12.1), Dendsort (0.3.4), multcomp (1.4-20), and SPSS (25.0)

For manuscripts utilizing custom algorithms or software that are central to the research but not yet described in published literature, software must be made available to editors and reviewers. We strongly encourage code deposition in a community repository (e.g. GitHub). See the Nature Portfolio guidelines for submitting code & software for further information.

Data

Policy information about availability of data

All manuscripts must include a data availability statement. This statement should provide the following information, where applicable:

- Accession codes, unique identifiers, or web links for publicly available datasets
- A description of any restrictions on data availability
- For clinical datasets or third party data, please ensure that the statement adheres to our policy

Microarray data has been deposited in GSE45291 and GSE121239. Antibody sequences from SLE-derived monoclonal antibodies were obtained from published information (references 6, 50 and 51).

Human research participants

Policy information about studies involving human research participants and Sex and Gender in Research.

Reporting on sex and gender	Both female and male participant included, as described in Supplementary Table 1
Population characteristics	Characteristics are included in Supplementary Table 1
Recruitment	All human samples used in this study were already collected and stored from previous studies. This included the analysis of sera from 62 healthy controls and 158 SLE patients from the "Study of biological Pathways, Disease Activity and Response markers in patients with Systemic Lupus Erythematosus" (SPARE) published in 2015 (Ref. 59). SPARE was a prospective observational study performed at the Lupus Center at Johns Hopkins. Following informed consent, adult patients were eligible if they were aged 18–75 years old and met the definition of SLE as defined by the revised American College of Rheumatology classification criteria.
Ethics oversight	All samples were obtained under approval by the Institutional Review Boards at the Johns Hopkins University School of Medicine and Emory University School of Medicine.

Note that full information on the approval of the study protocol must also be provided in the manuscript.

Field-specific reporting

Please select the one below that is the best fit for your research. If you are not sure, read the appropriate sections before making your selection.

☒ Life sciences ☐ Behavioural & social sciences ☐ Ecological, evolutionary & environmental sciences

For a reference copy of the document with all sections, see nature.com/documents/nr-reporting-summary-flat.pdf

Life sciences study design

All studies must disclose on these points even when the disclosure is negative.

Sample size	No formal power calculations were performed. The study included the analysis of stored samples and clinical data from 158 SLE patients from the "Study of biological Pathways, Disease Activity and Response markers in patients with Systemic Lupus Erythematosus" (SPARE) published in 2015 (Ref. 61). Since samples and data were already collected, the sample sized cannot change.
Data exclusions	No exclusions
Replication	1. cDNA encoding mature DNase1L3 was cloned only once and the sequence was confirmed only once. No more replication was required because the sequence was correct.<br>2. Expression of DNase1L3 by TNT T7 Quick Coupled Transcription/Translation (Promega) or PUREexpress was performed every time that was needed and the expression was always replicated.<br>3. Assays to screen for anti-DNase1L3 antibodies by immunoprecipitation were established and replicated at least 5 times before the full screen of the control and SLE serum.<br>4. Plasmids encoding monoclonal antibodies were cloned only once and their sequences were confirmed only once. No more replication was required because the sequences were correct.<br>5. Production of recombinant monoclonal antibodies using cells lines was performed in at least 5 different occasions with similar results.<br>6. To establish the optimal conditions to quantify DNase1L3 activity, we tested different final volumes, buffers, magnesium and calcium concentrations, number of purified cell nuclei, enzyme concentrations, BSA amounts, incubation time, and LM-qPCR assays. Once an optimal assay was established, it was replicated in at least 5 different occasions before its use in the final experiments.<br>7. The conditions to establish the in-house magnetic bead-based immunoassay used for monoclonal EC50s were defined using different assays (including different wash and blocking buffers). Once the optimal assay was established, it was replicated in at least 5 different occasions before its use in the final experiments.
Randomization	The study included the analysis of stored samples and clinical data from 158 SLE patients from the SPARE cohort published in 2015 (Ref. 61). In addition, the study included the functional analysis of previously cloned monoclonal antibodies. Therefore, randomization was not required.
Blinding	Since the nature of the study is observational blinding was not relevant.

Behavioural & social sciences study design

All studies must disclose on these points even when the disclosure is negative.

Study description	Not relevant for the study.

Research sample	Not relevant for the study.
Sampling strategy	Not relevant for the study.
Data collection	Not relevant for the study.
Timing	Not relevant for the study.
Data exclusions	Not relevant for the study.
Non-participation	Not relevant for the study.
Randomization	Not relevant for the study.

Ecological, evolutionary & environmental sciences study design

All studies must disclose on these points even when the disclosure is negative.

Study description	Not relevant for the study.
Research sample	Not relevant for the study.
Sampling strategy	Not relevant for the study.
Data collection	Not relevant for the study.
Timing and spatial scale	Not relevant for the study.
Data exclusions	Not relevant for the study.
Reproducibility	Not relevant for the study.
Randomization	Not relevant for the study.
Blinding	Not relevant for the study.

Did the study involve field work? ☐ Yes ☒ No

Reporting for specific materials, systems and methods

We require information from authors about some types of materials, experimental systems and methods used in many studies. Here, indicate whether each material, system or method listed is relevant to your study. If you are not sure if a list item applies to your research, read the appropriate section before selecting a response.

Materials & experimental systems

n/a	Involved in the study	
☐	☒	Antibodies
☐	☒	Eukaryotic cell lines
☒	☐	Palaeontology and archaeology
☒	☐	Animals and other organisms
☒	☐	Clinical data
☒	☐	Dual use research of concern

Methods

n/a	Involved in the study	
☒	☐	ChIP-seq
☒	☐	Flow cytometry
☒	☐	MRI-based neuroimaging

Antibodies

Antibodies used	Horseradish peroxidase (HRP)-conjugated goat anti-human IgG antibody (Jackson Immunoresearch, Code: 109-035-088)
Validation	ELISA

Eukaryotic cell lines

Policy information about cell lines and Sex and Gender in Research

Cell line source(s)	293T (These cells have been in the lab for the last ~25 years, a gift from a neighbor lab at Hopkins. The original source is unknown).<br>Expi293F cells. Thermo Fisher Scientific. Catalogue number A14527.<br>ExpiCHO-S cells. Thermo Fisher Scientific. Catalogue number A29127.
Authentication	The cell lines were authenticated by the manufacturer (Thermo Fisher Scientific).
Mycoplasma contamination	Expi293F and ExpiCHO-S cell lines were not tested for mycoplasma contamination. The manufacturer (Thermo Fisher Scientific) certified that the cell lines were Mycoplasma free.<br>293T cells were tested ~10 years ago before doing frozen aliquots. The 293T cells are from this batch of frozen cells.
Commonly misidentified lines<br>(See ICLAC register)	None used
